# mRNA vaccines for HBV: Mechanisms, preclinical advances, and therapeutic clinical progress

**DOI:** 10.1016/j.omtn.2025.102819

**Published:** 2025-12-29

**Authors:** Nadezhda A. Pechnikova, Maria Zafeiriou-Chatziefraimidou, Malamati Poimenidou, Chrysi Patsidou, Ioannis Iliadis, Yulia V. Ostankova, Alexey V. Yaremenko

**Affiliations:** 1 Elpida BioPharm P.C., 54636 Thessaloniki, Greece; 2 Laboratory of Chemical Engineering A’, Department of Chemical Engineering, Faculty of Engineering, Aristotle University of Thessaloniki, 54636 Thessaloniki, Greece; 3 Saint Petersburg Pasteur Institute, Saint Petersburg 197101, Russia; 4 School of Medicine, Aristotle University of Thessaloniki, 54124 Thessaloniki, Greece; 5 1st Department of Ophthalmology, AHEPA University Hospital, Aristotle University of Thessaloniki, Thessaloniki, Greece; 6 MSc in Ocular Surgery, School of Medicine, Aristotle University of Thessaloniki, Thessaloniki 54124, Greece

**Keywords:** MT: Delivery Strategies, mRNA vaccines, hepatitis B virus, functional cure, therapeutic vaccination, self-amplifying RNA

## Abstract

Hepatitis B virus (HBV) remains a leading cause of cirrhosis and hepatocellular carcinoma, while functionally curative therapies remain scarce. Durable remission is hindered by the persistence of covalently closed circular DNA (cccDNA) and viral genome integration, both of which contribute to impaired immune responses. mRNA-based technologies provide a versatile platform for prevention and treatment, owing to their rapid development cycle and intrinsic immunostimulatory properties. In preclinical HBV-carrier mouse models, lipid-nanoparticle (LNP) mRNA vaccines encoding hepatitis B surface antigen (HBsAg) or polyvalent Ags have achieved HBsAg clearance and HBV-DNA reduction, outperforming protein-based comparators. Clinically, two phase I therapeutic trials have been initiated to date. An ARCUS nuclease delivered as LNP-mRNA entered first-in-human testing in 2025, with only preliminary safety data available from a small patient cohort and no published efficacy data. Similarly, HBx-biased mRNA vaccines, such as WGc-0201, have recently entered early clinical evaluation without reported clinical efficacy. Despite these advances, several challenges impede effective therapy, including innate-immune overactivation, HBV genotypic diversity, and the need for rational therapeutic combinations. This review summarizes current preclinical findings and emerging clinical evidence and outlines future strategies toward durable viral control and effective prophylaxis, including self-amplifying or cyclic RNAs, receptor-targeted LNPs, and multimodal therapeutic regimens.

## Graphical abstract

**Figure:**
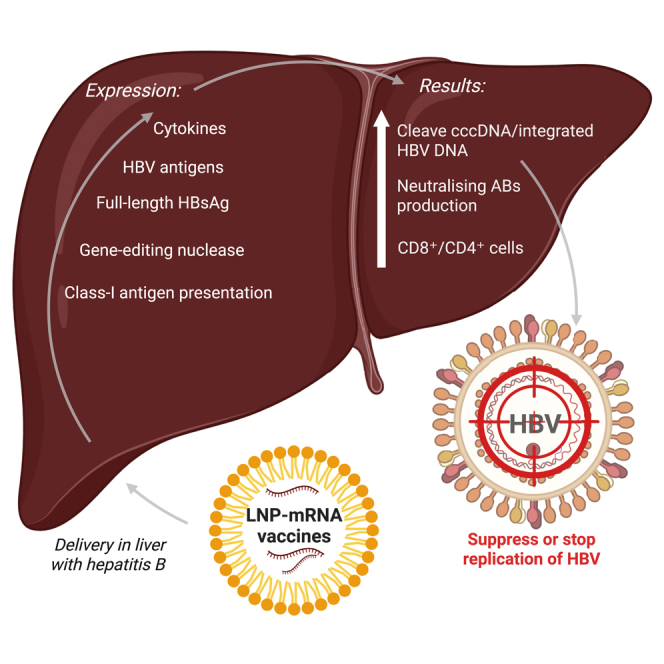

## Teaser

This review summarizes advances in LNP-mRNA platforms for hepatitis B, spanning antigen-encoding therapeutic vaccines and mRNA-delivered gene editors targeting cccDNA or integrated HBV DNA. Pechnikova and colleagues discuss preclinical efficacy, early clinical trials, mechanistic immune reprogramming, and key translational hurdles, highlighting the combination regimens required for a durable functional cure.

## Introduction

Although the hepatitis B virus (HBV) was identified only in the latter half of the 20th century, phylogenetic evidence shows that it has circulated among humans for thousands of years^1^ —and it remains a major global health challenge.^2^ According to the World Health Organization (WHO), about 254–316 million people live with chronic HBV infection worldwide,^3^^,^^4^^,^^5^^,^^6^^,^^7^ and roughly 1.2 million new infections occur each year. HBV is primarily transmitted via exposure to infected blood or body fluids, either through skin breaches or mucosal surfaces.^6^^,^^8^ An additional and particularly significant route of transmission is perinatal, occurring from mother to child during pregnancy, delivery, or postnatally via the placenta, birth canal, or breastfeeding. Infected individuals not only remain highly contagious but also face a markedly increased risk of progressive liver damage, such as fibrosis and cirrhosis.^7^^,^^9^^,^^10^ Furthermore, HBV can integrate fragments of viral DNA into the host genome, which is associated with the development of hepatocellular carcinoma (HCC);^11^ approximately 25% of people with chronic HBV infection are at risk of premature death.^7^^,^^9^^,^^10^

Current antiviral therapies for HBV infection, including interferon (IFN) formulations and nucleos(t)ide analogs (NAs), have significant clinical limitations. They primarily suppress viral replication but are not curative and require survival therapy due to the formation of persistent covalently closed circular DNA (cccDNA) reservoirs in the human body. In addition, IFN-based regimens, such as IFN-alpha (IFN-α)^12^ and pegylated IFNs (PEG-IFNs),^13^^,^^14^ are associated with poor rates of HBV e-antigen (HBeAg) seroconversion and require frequent dosing, which can lead to poor patient adherence.^6^^,^^15^ In addition, these treatments often cause influenza-like symptoms, further reducing tolerability.^12^^,^^13^^,^^14^ Drugs such as lamivudine,^16^ adefovir,^17^ telbivudine,^18^ and entecavir^19^ also have substantial drawbacks. Many of these agents are prone to the development of viral resistance—lamivudine and telbivudine, for example, are associated with high resistance rates, while adefovir is characterized by nephrotoxicity and a low genetic barrier to resistance.^20^ Long-term use of tenofovir^21^ can result in bone loss, and tenofovir disoproxil fumarate^22^ may cause varying degrees of dyslipidemia. Compliance with long-term entecavir therapy is often suboptimal, compromising virologic suppression. Moreover, concomitant treatment for non-HBV-related illnesses, such as the selective T cell costimulatory modulator abatacept, may increase the risk of HBV reactivation and worsen the course of the disease.^23^

Alternative approaches, such as recombinant protein-based, adenoviral vector-based, and DNA-based vaccines, have demonstrated varying degrees of success in hepatitis B surface Ag (HBsAg) seroclearance and antibody (Ab) seroconversion, yet each method presents significant limitations. For example, recombinant protein-based vaccines often induce weak immunogenicity, requiring multiple booster doses and adjuvants to enhance immune responses. Their reliance on humoral immunity limits their ability to generate a strong cytotoxic T lymphocyte (CTL) response, which is crucial for viral clearance. Adenoviral vector-based vaccines improve T cell responses, but concerns regarding pre-existing immunity to viral vectors, potential inflammatory reactions, and limited durability of immune responses hinder their widespread adoption. DNA-based vaccines that directly encode HBV Ags have a few limitations associated with low transfection efficiency, weak Ag expression, and insufficient immunogenicity in humans.^24^^,^^25^ Also, of great importance in the development of new methods of therapy for HBV is the fact that, despite the combined administration of HBV immunoglobulin and vaccination against HBV, 10%–30% of infants born to mothers with a high viral load can still become infected.^26^ Collectively, these issues highlight the urgent need for novel, more effective, and safer therapeutic strategies for chronic HBV infection.

A new promising strategy for HBV therapy is the development of messenger RNA (mRNA)-based vaccines that encode HBV Ags to stimulate the immune system and induce protective or therapeutic responses against HBV.^27^ These vaccines could exhibit superior immunogenicity, eliciting both strong humoral and cellular immune responses.^27^^,^^28^^,^^29^ Moreover, unlike traditional approaches, mRNA vaccines provide intrinsic adjuvant properties, enhancing innate immune activation, which may contribute to improved antiviral immunity. Furthermore, their rapid production, scalability, and adaptability position them as a promising strategy for future HBV vaccine development.^28^^,^^29^ Advances in *in vitro*-transcribed mRNA technology offer significant advantages over viral vector and DNA-based vaccines in terms of safety, cost-effectiveness, and manufacturing efficiency.^30^ Although only two phase I clinical trials investigating mRNA- or lipid-nanoparticle (LNP)-based therapies for HBV have commenced in 2025— Precision BioSciences Gene Editing - Hepatitis B Virus (PBGENE-HBV) (enrolled 3 patients) and WGc0201 (will enroll 9 patients)—currently, publicly available data remain limited. However, the surge in preclinical successes and announcements of new drugs points to the growing potential of mRNA technologies for both the prevention and treatment of HBV.

In this review, we examine mRNA-based approaches for HBV treatment and prevention. We provide an overview of vaccine development, preclinical and clinical trial progress and their challenges, and future prospects in this rapidly evolving field.

## Etiology and pathophysiology of HBV

### Virology

HBV belongs to the *Hepadnaviridae* family, a unique group of DNA viruses that replicate through an RNA intermediate and attacks liver cells. It is a ∼42 nm enveloped “Dane” particle: a partially double-stranded 3.2 kb DNA genome resides inside an icosahedral nucleocapsid, which in turn is wrapped by a lipid envelope studded with three surface-Ag isoforms—small (S), middle (M), and large (L) HBsAg glycoproteins.^31^^,^^32^^,^^33^ During natural infection, the envelope proteins are translated from the same *S* gene but from three in-frame start codons, yielding L-, M- and S-HBs in a regulated ratio. The L protein drives hepatotropism via binding the sodium taurocholate co-transporting polypeptide (NTCP)^34^ and its coreceptor, epidermal growth factor receptor.^35^^,^^36^ M optimizes particle release and immunogenicity, and S ensures efficient envelopment and immune distraction. Together, they balance infectivity with immune evasion, explaining why modern vaccine strategies are beginning to incorporate preS1/2 sequences to broaden neutralizing breadth beyond the classical S-only formulations.^37^^,^^38^ Other candidate HBV-binding proteins at the cell surface have been described—fibronectin and the hepatic asialoglycoprotein receptor.^39^ Once inside the hepatocyte, HBV converts its relaxed circular DNA (rcDNA) into cccDNA that serves as a stable template for transcription. Replication proceeds by reverse transcription of an RNA pre-genome within cytoplasmic nucleocapsids, a strategy more commonly associated with retroviruses.^31^^,^^32^ Although HBV’s reverse-transcription step is error-prone, the virus accrues changes at only ∼1 × 10^−5^ substitutions per base each year.^40^^,^^41^ Even at this modest pace, it has diversified into ten genotypes (A–J), each ∼8% divergent across the genome, and >40 recognized sub-genotypes.^42^ Added to this is a steadily expanding catalog of immune-escape mutations in the surface “a” determinant (amino acids [aa] 124–147 of HBsAg—the main B cell epitope targeted by neutralizing Abs), core, and X proteins (HBx, the 154-amino-acid product of the HBV X open reading frame). From a kinetic perspective, HBV is remarkably productive: an infected liver can release 10^10^–10^12^ virions each day, even though individual virions have a short half-life of only 2–3 days in circulation. This prodigious output, coupled with cccDNA persistence and genomic integration, explains why chronic HBV infection is so difficult to eradicate and why novel approaches—such as mRNA-based therapeutic vaccines that elicit potent antiviral immunity—are now being pursued.^31^^,^^32^

### Pathophysiology

**Figure 1:**
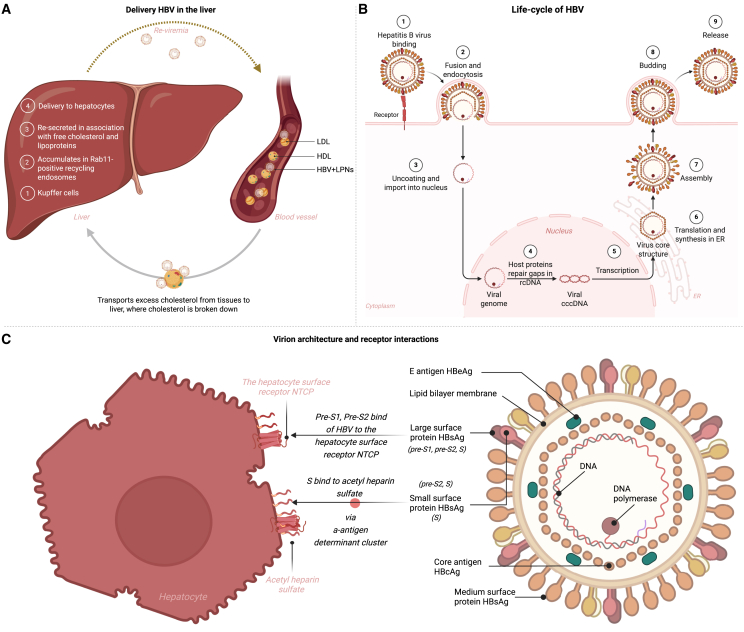
Pathways of HBV hepatotropism, replication, and host-cell engagement (A) Delivery of HBV to the liver via ApoE-lipoprotein detour. After entering the bloodstream, HBV virions are first captured by liver-resident macrophages and accumulate in Rab11-positive recycling endosomes. They are then re-secreted bound to ApoE-enriched lipoproteins and free cholesterol, hitchhiking the reverse-cholesterol-transport loop to reach hepatocytes with high efficiency. (B) Canonical intracellular life cycle of HBV. (C) Virion architecture and receptor interactions. The Dane particle’s lipid envelope displays the large (preS-1 + preS-2 + S), middle (preS-2 + S), and small (S) HBsAg isoforms. The preS-1 and preS-2 regions bind the NTCP entry receptor, while the S domain engages heparan sulfate proteoglycans via the a-antigen determinant cluster.

Recent findings reveal an additional mechanism by which HBV enhances its hepatotropism: association with lipoproteins, particularly Alloprotein E (ApoE)-enriched particles, and hijacking of the reverse cholesterol transport pathway (Figure 1A).^43^ In this route, HBV is first taken up by liver-resident macrophages (Kupffer cells), where it accumulates in Rab11-positive recycling endosomes instead of undergoing lysosomal degradation.^43^ The virus is then re-secreted in association with free cholesterol and lipoproteins, allowing its efficient delivery to hepatocytes. This transcytosis-like pathway, exploiting physiological lipid transport mechanisms, ensures highly specific and efficient targeting of hepatocytes and may explain the virus’s remarkable infectivity—even a single virion can establish infection (Figure 1B).^43^ The initial step in viral entry into hepatocytes is mediated by a low-affinity interaction between HBsAg—a glycoprotein on the viral envelope—and heparan sulfate proteoglycans located on the hepatocyte surface within the space of Disse (Figure 1C). This facilitates viral retention in proximity to hepatocytes. Subsequent high-affinity binding to the NTCP—a bile acid transporter expressed exclusively on the basolateral membrane of hepatocytes—enables viral internalization and initiates infection. After the insertion of the virus into a hepatocyte, the viral genome is transported into the nucleus and is converted by ligation into cccDNA, a stable form that plays a key part in the maintenance of chronic HBV. Subsequently, pregenomic RNA (pgRNA) is transferred from the nucleus to the cytoplasm and forms the template for reverse transcription into circular double-stranded rcDNA within the nucleocapsid. The pgRNA also acts as a mRNA for the synthesis of viral proteins, including the core protein and polymerase. HBV nucleocapsids enclosing newly formed rcDNAs are egressed from the hepatocyte through the secretory pathway or go through recycling pathways. Only about 10% of these new virions contain a double-stranded linear genome that is proficient forerunners for integration of the HBV genome into the host cell’s DNA, which contributes to viral persistence.^8^^,^^44^^,^^45^

### Immunopathogenesis and clinical outcomes of acute and chronic HBV infection

**Figure 2:**
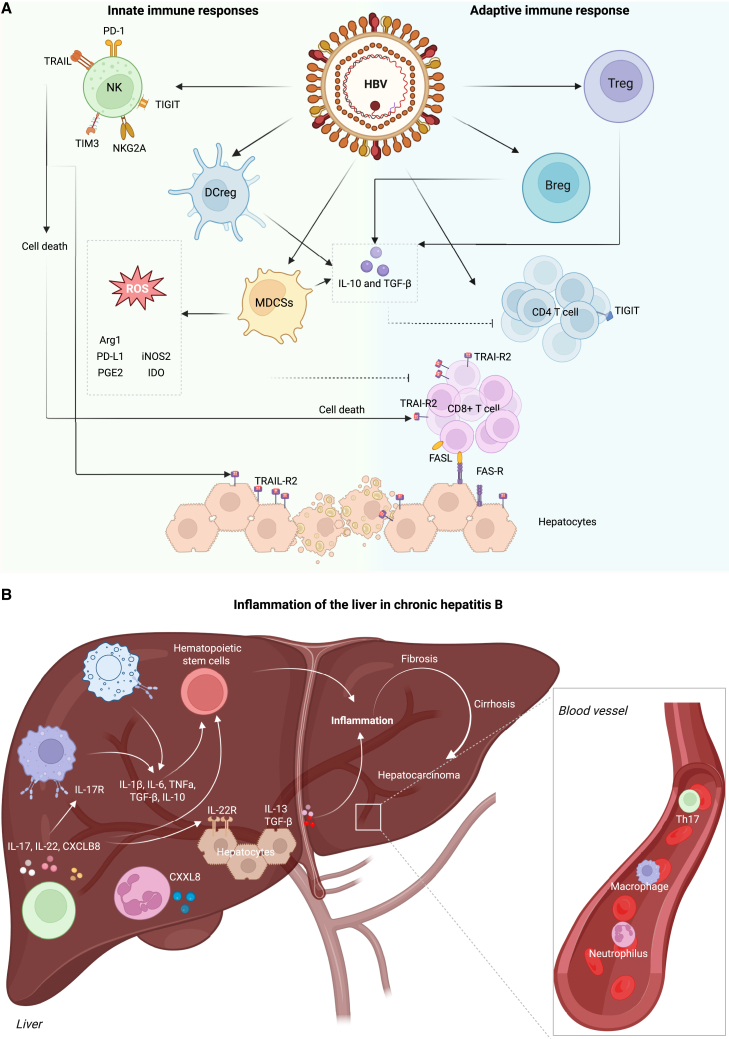
Immunological mechanisms and liver inflammation in chronic HBV (A) The innate and adaptive immunity in chronic HBV. The virus promotes immune tolerance by stimulating regulatory dendritic cells (DCreg), myeloid-derived suppressor cells (MDSCs), and the production of immunosuppressive cytokines-interleukin 10 (IL-10) and transforming growth factor β (TGF-β). These mediators inhibit effective T cell responses and enhance expression of inhibitory molecules such as PD-1, T-cell immunoglobulin and munich-domain containing 3 (TIM-3), T-cell immunoreceptor with Ig and ITIM domains (TIGIT), and natural killer group 2 member A (NKG2A) on NK and CD8^+^ T cells, impairing cytotoxic activity. MDSCs release immunosuppressive factors, including arginase-1 (Arg1), PD-L1, inducible nitric oxide synthase (iNOS2), prostaglandin E2 (PGE2), idoleamine 2,3-dyoxigenase (IDO1), and reactive oxygen species, contributing to T cell dysfunction and hepatocyte survival. Meanwhile, Treg and Breg cells further suppress effector T cell activity. Apoptotic pathways mediated by FAS/FAS-L and TRAIL/TRAIL-R2 lead to hepatocyte and immune cell death, maintaining persistent infection. (B) Chronic inflammation of the liver in HBV infection and progression to fibrosis and cancer. The hepatic microenvironment in chronic HBV is characterized by persistent inflammation driven by pro-inflammatory cytokines (IL-1β, IL-6, TNFα), chemokines such as chemokine (CXC motif) ligand 8 (CXCL8), and fibrogenic mediators—IL-13 and TGF-β. Hepatic stellate cell activation and cytokine production drive progression from inflammation to fibrosis, cirrhosis, and eventually HCC. Th17 cells, neutrophils, and macrophages infiltrate the liver and release cytokines such as IL-17, IL-22 and CXCL8, exacerbating the inflammatory cascade. Hematopoietic stem cells contribute to immune cell recruitment and activation. The interplay of these pathways sustains chronic liver damage and immune dysfunction.

HBV is generally considered a non-cytopathic virus, meaning it does not directly induce hepatocyte death. Instead, the liver inflammation and tissue damage observed during acute and chronic HBV infection are primarily the result of host immune-mediated responses targeting infected hepatocytes. The clinical trajectory of HBV—ranging from spontaneous viral clearance to persistent chronic hepatitis—is largely determined by the magnitude, timing, and coordination of the host’s innate and adaptive immune responses (Figure 2A).^46^

Clinical signs of acute HBV infection in adults become apparent when the virus is detectable in the bloodstream and the level of HBV DNA in the serum rises rapidly, before typically decreasing. Presumably HBV clearance occurs predominantly by a non-cytolytic mechanism in which virus-specific CD8^+^ T cells secrete IFN-gamma (IFN-γ) and tumor necrosis factor (TNF), achieving viral suppression without appreciable immune-cell infiltration or overt hepatocyte destruction.^8^^,^^47^ Besides virus specific CD8+ T cells, during the acute resolving phase of HBV, CD4+ T cells support CD8+ T cells in effective viral clearance, and B cells produce and release neutralizing Abs against HBsAg (anti-HBs). Clinically apparent acute HBV is uncommon in newborns,^48^ yet infection acquired in the first year of life becomes chronic in more than 90% of cases due to the immaturity of the infant immune system.^49^ At this age, incomplete development and poor coordination between innate and adaptive immunity significantly impair viral clearance, resulting in a high risk that early-life HBV infection will progress to chronic HBV.^8^^,^^44^^,^^46^^,^^50^

A major contributor to viral persistence is the formation and maintenance of cccDNA within hepatocyte nuclei. This stable viral reservoir acts as a template for continuous viral replication, while remaining shielded from immune detection. Additionally, the excessive production of HBsAg acts as a decoy, further suppressing host immune responses.^45^ Impairment in the function of key immune effectors—including natural killer (NK) cells, Kupffer cells, and cytotoxic lymphocytes—leads to suboptimal cytokine production and promotes an immunotolerant hepatic environment, reducing inflammation and facilitating ongoing viral replication.^50^^,^^51^^,^^52^ Even in chronic infection, HBV-specific T cells infiltrate the liver and attempt to control the virus through cytolytic and non-cytolytic mechanisms. However, viral immune escape mutations and persistent Ag exposure drive T cell exhaustion—a state characterized by functional impairment and loss of proliferative capacity. This exhaustion is a hallmark of chronic HBV, affecting both CD4^+^ and CD8^+^ virus-specific T cell populations, and is considered the central mechanism underlying immune failure in chronic HBV.^53^^,^^54^^,^^55^

### Fibrosis and cirrhosis in chronic HBV

In chronic HBV, the persistent immune-mediated response to viral Ags leads to a repetitive cycle of hepatocyte injury and regeneration (Figure 2B). This prolonged interaction between the immune system and viral components results in the accumulation of extracellular matrix in areas undergoing repair, ultimately contributing to the progressive development of hepatic fibrosis.^56^ For example, over 40% of individuals with chronic HBV infection who do not receive treatment eventually develop liver cirrhosis.^57^^,^^58^^,^^59^ As hepatocytes are gradually lost due to ongoing inflammation and immune attack, the functional capacity of the liver declines proportionally^60^ and causes a negative impact on health. Moreover, the severity and progression of fibrosis in chronic HBV are influenced by the phase of infection, serum levels of alanine aminotransferase (ALT), HBV DNA viral load, and sex.^61^

As fibrosis progresses, the liver can advance to cirrhosis, the terminal stage of chronic HBV injury.^8^^,^^45^^,^^56^ Cirrhosis is defined by widespread extracellular matrix deposition that reorganizes the vasculature and replaces normal lobular architecture with regenerative nodules encased in fibrotic septa. Blood is shunted through neo-vessels along these septa, bypassing functional hepatocytes and precipitating portal hypertension, hepatic insufficiency, and, ultimately, liver failure.^62^^,^^63^ The same injury-repair cycle that drives fibrosis also promotes oncogenesis: 70%–90% of patients with HBV-related cirrhosis eventually develop HCC.^64^^,^^65^^,^^66^^,^^67^ Multiple, still-unfolding mechanisms underlie HBV oncogenicity, including persistent high-level viral replication, genotype-specific viral factors, integration of viral DNA into host chromosomes, expression of viral oncoproteins, and a chronic inflammatory milieu that accelerates hepatocyte turnover and mutagenesis.^11^^,^^64^ For these reasons, HBV remains a leading global cause of cirrhosis, HCC, and liver-related mortality.

### Problem current prevention of HBV

Despite the availability of a highly efficacious recombinant vaccine for more than three decades, the global program to prevent HBV infection still falls short of public-health targets. According to the WHO, 3 infant doses now reach roughly 84% of birth cohorts worldwide.^68^ The time-critical birth dose that blocks perinatal transmission is administered to fewer than one-half of newborns overall—and to only 17%–18% in sub-Saharan Africa—because many deliveries occur outside health facilities, cold-chain capacity is patchy, and supply chains are fragile. Field studies show that storing the heat-stable monovalent vaccine outside the conventional cold chain can more than quadruple on-time birth-dose uptake in remote settings, but such adaptations are not yet widely implemented.^68^

Coverage gaps persist beyond infancy. In the United States, for example, just 34% of adults have ever completed an HBV vaccine series, with even lower uptake among older or socio-economically disadvantaged groups, illustrating how multi-dose schedules and reimbursement hurdles impede adult protection. Biological limitations compound programmatic ones: 5%–10% of immunocompetent vaccinees fail to produce protective anti-HBs.^68^ And immune-escape surface-Ag variants with a specific mutation in the HBV surface protein (G145R) are now detectable in about 1% of global sequence databases, with overall escape-mutation frequencies exceeding 10% in some HBV genotypes.^69^

Moreover, mother-to-child transmission has not been fully eliminated. This is because timely birth dosing alone cannot protect infants born to mothers with very high viraemia. WHO-recommended tenofovir prophylaxis remains uneven in settings with limited access to antenatal viral load testing and drug procurement.^70^

Taken together, the shortcomings of current prevention—insufficient birth-dose coverage, low adult uptake, vaccine non-response, emergence of escape mutants, and incomplete maternal prophylaxis—underscore the need for next-generation tools, including single-dose thermostable vaccines, simplified adult schedules, and novel platforms such as mRNA, to close the residual prevention gap.

### Problem current treatment of acute HBV

Treatment of acute HBV is almost entirely supportive. Patients usually receive rest, fluid replacement, and drugs to control symptoms. Clinical trials with lamivudine^71^ and pooled small studies^72^ have not shown consistent benefit from antiviral drugs. No available medicines reliably shorten the illness, speed up HBsAg clearance, or prevent progression to chronic infection.

### Problem current treatment of chronic HBV

At present, the therapeutic objective in chronic HBV is not complete viral eradication but rather the reduction of the risk for hepatic decompensation and the development of HCC. The achievement of these goals relies primarily on effective suppression of HBV replication, which facilitates normalization of serum ALT and attenuation of hepatic necroinflammatory activity.^73^ Currently, 7 antiviral agents are approved for the treatment of chronic HBV infection in the USA, classified into two main categories: IFNs and NAs.^74^

The first class is IFNs, which can exhibit both immunomodulatory and antiviral properties. In the context of chronic HBV, PEG-IFN-α is the primary agent used, administered via subcutaneous injection. PEG-IFN-α has been shown to induce HBeAg seroconversion—that is, the loss of HBeAg with the appearance of anti-HBe Abs—at higher rates compared to certain NA therapies.^75^ Additionally, it has demonstrated efficacy in reducing the long-term risk of disease progression, including the development of cirrhosis and HCC, especially when compared to untreated patients.^76^ However, treatment response to IFNs is variable and often suboptimal. Moreover, the adverse effect profile of PEG-IFN-α, such as cytopenia, autoimmune thyroid disorders, insomnia, and depression, may limit its use.^77^

The 5 NAs available in the USA—lamivudine, adefovir, entecavir, tenofovir disoproxil, and tenofovir alafenamide—act to reduce HBV DNA viral load and improve hepatic histology. Thereby, they help to slow the progression of liver disease and reduce the incidence of HCC in patients with advanced liver disease. Additionally, some NAs, such as lamivudine and tenofovir disoproxil, are considered potentially safe for use during pregnancy. They have been shown to reduce the risk of vertical transmission of the virus to the newborn.^75^ However, preclinical studies have raised concerns regarding the potential carcinogenicity of entecavir, as it has shown cytotoxic effects in human lymphocytes and tumor formation *in vivo*, such as in rats. Consequently, entecavir is not recommended for use in women of childbearing age or in pediatric patients.^73^ Also, therapy with lamivudine, adefovir, entecavir, tenofovir disoproxil, and tenofovir alafenamide is generally associated with mild to moderate adverse effects, such as fatigue, dizziness, headaches, and nausea.^78^^,^^79^ An additional major issue with this class of drugs is the development of resistant HBV strains due to the virus’s ability to develop tolerance after prolonged use. Although more recently developed NAs have much lower rates of resistance development, it has not been entirely eliminated. In terms of the effectiveness of this therapeutic class, tenofovir disoproxil and entecavir have been shown to be more effective than lamivudine and adefovir.^45^

In additional to these, the combination of the two aforementioned treatment categories has been investigated. While the addition of PEG-IFN to NAs in certain cases has shown improvement in HBsAg loss, generally, this combination therapy has not demonstrated more beneficial outcomes compared to monotherapy.^80^^,^^81^ The loss of HBsAg is associated with suppression of HBV DNA and represents the ideal outcome of antiviral therapy. However, this occurs in only 3%–11% of patients treated with IFN and in 1%–12% of patients receiving NAs for 5 to 7 years.^45^^,^^82^ Consequently, the majority of patients initiating treatment with NAs remain on long-term therapy.^83^^,^^84^ Discontinuation of therapy may be considered if viral suppression and HBsAg loss are achieved. However, this remains a controversial issue due to the risk of relapse or development of HCC. Moreover, for patients without HBsAg loss, discontinuation of treatment may be considered due to the potential long-term toxic effects, the cost of medications, and the risk of resistance development.

## Mechanism of mRNA Therapy for HBV

The mechanism of mRNA-based therapy for HBV exploits two linked biological principles.

First, the delivery of Ag-coding mRNA to Ag-presenting cells and hepatocytes drives *de novo* synthesis of HBV proteins (e.g., HBsAg, HBc, HBx) inside the cytosol.^85^

The translated Ags are processed through the endogenous class I major histocompatibility complex (MHC I) pathway and then secreted. This secretion simultaneously activates innate sensors, including toll-like receptor 7/8 (TLR7/8) and retinoic acid-inducible gene I (*RIG I*), and generates potent polyfunctional CD4^+^ and CD8^+^ T cell responses together with neutralizing anti-HBs Abs. These immune effects have cleared HBsAg and suppressed viraemia in HBV-carrier mice and are now entering human trials.^28^^,^^86^

Second, the same delivery platform can shuttle mRNA that encodes a sequence-specific nuclease (ARCUS) into hepatocytes. Once translated and imported into the nucleus, the nuclease cuts cccDNA and integrated HBV sequences, thereby extinguishing the templates that sustain chronic infection.^87^^,^^88^ Together, these modes—immune re-education plus direct genetic excision of viral reservoirs—define the mechanistic foundation of mRNA therapy for HBV and distinguish it from traditional protein vaccines or polymerase inhibitors, offering a path toward true functional cure rather than lifelong suppression.

## mRNA-based vaccines in preclinical trials

Animal models are indispensable for ranking HB mRNA candidates, yet each platform captures only a subset of the human disease. Classical HBV-transgenic lines of mice (e.g., Alb-HBs) and plasmid hydrodynamic injection as pAAV-HBV1.2 into mouse liver cells express viral proteins from an integrated or episomal transgene but completely sidestep NTCP-dependent viral entry and never establish authentic cccDNA. Also, adeno- and adeno-associated virus (AAV)-driven systems such as rAAV8-HBV1.3 introduce more persistent viraemia and partially mimic cccDNA, but HBV transcription remains under the control of artificial promoters embedded in the viral backbone.^89^ In these case, gene-editing or mRNA-based approaches that delete or disrupt the HBV expression cassette primarily eliminate an ectopic, promoter-driven transgene, not the full spectrum of authentic cccDNA reservoirs nor the capacity for ongoing reinfection. As a result, such strategies can give a misleading impression of sterilizing cure in preclinical systems, whereas in natural chronic infection, the liver would remain vulnerable to reinvasion by circulating virus.^90^

Thus, any strategy that merely silences or excises the transgene (small interfering RNA [siRNA], mRNA-encoded nucleases) can look curative even though, in patients, freshly produced virions would continually reseed naive hepatocytes.^28^^,^^89^

To model genuine entry and replication—including cccDNA formation—researchers turn to human-liver chimeric mice. These animals permit authentic HBV infection in human hepatocytes, allowing quantification of LNP delivery to the target cell. However, they are immunodeficient, precluding assessment of vaccine-driven T and B cell reconstitution.^89^^,^^91^ Dual humanized mice that graft both hepatocytes and a human immune system partly solve this but remain expensive and variable. Large-animal surrogates (duck, woodchuck, tree shrew) provide chronic hepadnavirus infection with intact immunity and inexpensive husbandry, yet genotype differences complicate interpretation, and reagent availability is poor.^89^ Chimpanzees recapitulate the full human life cycle and immune pathology but are now largely inaccessible for ethical reasons. Although delivery barriers are easiest to study in hepatocyte-only chimeras, immunogenicity and cytokine-driven reactogenicity require immunocompetent hosts; consequently, regulators increasingly request data from both settings before first-in-human dosing.^89^^,^^90^^,^^91^^,^^92^

These gaps complicate translation and lead to candidates that cure mice often underperforming in woodchucks or chimpanzees. The field now pairs AAV models with humanized-liver mice or *ex vivo* liver-chimeric cultures to obtain more predictive readouts.

**Figure 3:**
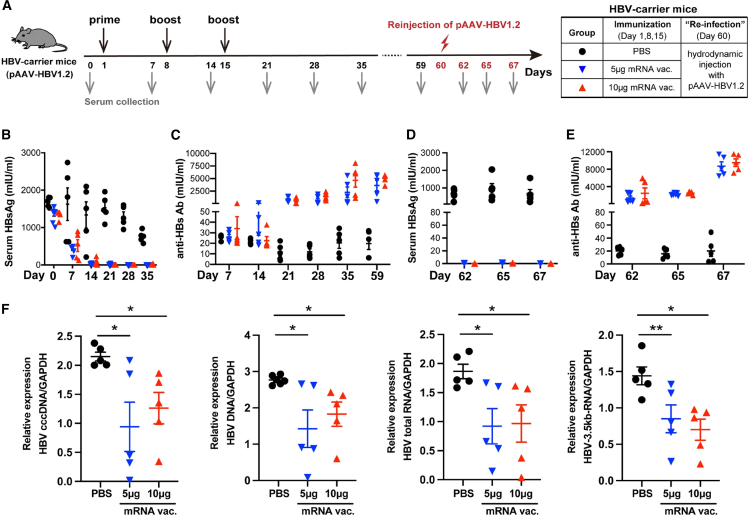
HBV mRNA–LNP vaccination suppressed viral markers and induced seroconversion in the pAAV-HBV1.2 mouse model (A) Experimental scheme with PBS as control. (B and D) Decline of serum HBsAg over time, demonstrating antigen clearance in both vaccine groups. (C and E) Robust seroconversion (anti-HBs antibody development) following administration of the HBV mRNA–LNP vaccine. (F) Changes in hepatic HBV cccDNA, total HBV DNA, and total HBV RNA levels compared with controls. Data are expressed as mean ± SEM and were analyzed using two-tailed unpaired Student’s *t* tests; statistical significance: *p* ≤ 0.05 (∗), *p* ≤ 0.01 (∗∗), *p* ≤ 0.0001 (∗∗∗). In this model, HBV gene expression is driven by an artificial hepatocyte-specific promoter from an AAV-encoded 1.2× HBV genome, rather than from authentic cccDNA, and the system does not recapitulate ongoing viral reinfection. As a result, elimination or silencing of the transgene may overestimate the efficacy required to clear cccDNA and to prevent reinfection in chronically infected patients. Reproduced under the terms of the CC-BY license.^28^ 2024, Zhao et al., published by Springer Nature.

**Figure 4:**
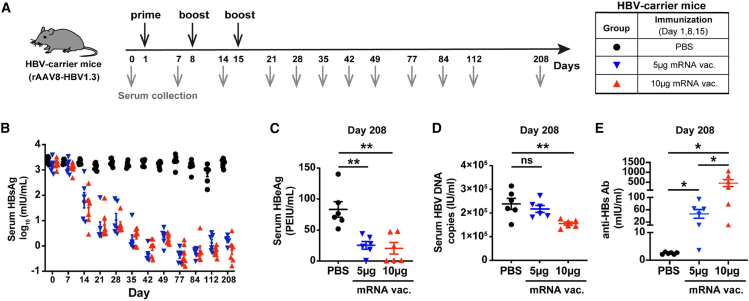
The HBV mRNA vaccine produced potent and sustained viral suppression and triggered robust seroconversion in rAAV8-HBV1.3 carrier mice (A) Experimental setup where controls were PBS. Serum samples were collected at multiple time points. (B) Serum HBsAg levels were monitored longitudinally. On day 208 post-treatment initiation, serum levels of (C) HBeAg, (D) HBV DNA, and (E) anti-HBs Abs were measured. Data represent the mean ± SEM and were analyzed by unpaired, two-tailed Student’s *t* tests (*p* ≤ 0.05 (∗), *p* ≤ 0.01 (∗∗)). PEIU: Paul-Ehrlich-Institute Unit. In this model, HBV gene expression is driven by an artificial hepatocyte-specific promoter from an AAV-encoded 1.2× HBV genome, rather than from authentic cccDNA, and the system does not recapitulate ongoing viral reinfection. As a result, elimination or silencing of the transgene may overestimate the efficacy required to clear cccDNA and to prevent reinfection in chronically infected patients. Reproduced under terms of the CC-BY license.^28^ 2024, Zhao et al., published by Springer Nature.

The focus of mRNA-based vaccines in preclinical trials is on vaccines that encode HBV Ags (core, polymerase, and preS2-S surface),^93^ a full-length HBsAg,^28^ 3 envelope proteins (L + M + S),^94^ N-terminal MHC-I signal peptide, and a C-terminal MHC-I trafficking domain (MITD).^95^ For example, to overcome the limitation of persistent immune tolerance primarily driven by HBsAg, a novel LNP-formulated mRNA vaccine was developed. This vaccine encodes three key HBV Ags—core, polymerase, and preS2-S surface (envelope)—with the aim of generating broad, polyfunctional T cell and Abs responses.^93^ Vaccination *in vivo* (AAV-HBV-transduced mice) generated strong Ag-specific, IFN-γ^+^/TNF-α^+^ CD8^+^ and CD4^+^ T cell populations and induced both anti-HBs and anti-HBe Abs. After 3 doses, 50% of mice showed 1.0–1.7 log_10_ IU mL^−1^ reductions in serum HBsAg; transient HBeAg declines mirrored these responders. Despite the serum effect, no treatment-related drop in intra-hepatic viremia or HBsAg-positive hepatocytes was detected, and ALT remained comparable to control. Thus, these data show that the multiAg vaccine was able to induce HBV-specific Abs and polyfunctional T cells in 50% of mice. However, the efficacy of the vaccine was modest, restricted to serum antigenemia, and did not translate into hepatic viral clearance, suggesting insufficient attack on the cccDNA reservoir. Presumably the use of three transcripts results in the formation of a larger overall mRNA packet, more epitopes, but a lower specific dose of HBsAg.^93^ In another study, the mRNA vaccine coding a full-length HBsAg was formulated in an LNP and delivered intramuscularly to chronically infected mice rendered HBsAg-positive via pAAV-HBV1.2 (Figure 3) or rAAV8-HBV1.3 (Figure 4) transduction.^28^ This choice was informed by several factors, including optimal display of conformational epitopes, concentrated Ag dosing to break B cell tolerance, subviral particles formation serving as a self-adjuvant, streamlined regulatory approval due to extensive clinical familiarity with HBsAg, and a direct serum biomarker (HBsAg decline) for evaluating vaccine efficacy in real time.^28^Injections of LNP-mRNA vaccine induced a precipitous fall in circulating HBsAg to the assay’s lower limit (∼0 mIU mL^−1^) (Figures 3B and 3D) and raised geometric-mean anti-HBs Ab titers to 3,624–4,804 mIU mL^−1^ (Figures 3C and 3E). Also, the intramuscular vaccination with full-length HBsAg mRNA-LNP lowered every intra-hepatic viral marker—cccDNA (Figure 3F), total HBV DNA, total HBV RNA, and 3.5-kb pgRNA—by roughly 40%–60% relative to controls (group treated with phosphate-buffered saline [PBS], indicating meaningful suppression of the hepatic viral reservoir. Concomitantly, there was robust virological suppression—serum HBV DNA decreased to ∼1.5 × 10^4^ copies mL^−1^, an order of magnitude lower than the 1.75× 10^5^ copies mL^−1^ observed in controls. Immunophenotyping showed marked recruitment and maturation of splenic Ag-presenting cells—including CD8α^+^ and CD103^+^ cDC1, CD11b^+^ cDC2 dendritic subsets, and macrophages—paralleled by expansion of Th1-polarized CD4^+^ and CD8^+^ T cells secreting IFN-γ or interleukin (IL)-2.^28^ In rAAV8-HBV1.3 carrier mice (Figure 4A), three weekly intramuscular doses of the full-length HBsAg mRNA-LNP vaccine produced a swift and lasting virological response: serum HBsAg plummeted by > 1 log_10_ within 5 weeks and remained at or below the detection threshold through day 208 (Figure 4B). By the same late time point, HBeAg levels had fallen by ∼60% (Figure 4C), and serum HBV DNA copies decreased by roughly one log in the 10 μg groups (Figure 4D). In high-dose groups, anti-HBs Ab reached ∼150 mIU mL^−1^, while controls remained Ab-negative (Figure 4E).^28^ These data demonstrate that this mRNA elicits coordinated humoral and cellular immunity capable of achieving sustained functional cure in stringent murine models of chronic hepatitis B, thereby justifying advancement to clinical development. Nevertheless, AAV based models generate low and unstable pools of authentic cccDNA; hence, while the downward shift is encouraging, its translational value is still limited.^28^

In another study, there was also an attempt to solve the problem related to functional cure of chronic HBV being thwarted by high circulating **HBsAg**.^94^ Because neutralizing epitopes reside in the pre-S1/S2 domains of the large surface protein (L-HBs), it was hypothesized that stably displaying all three envelope proteins (L + M + S) on host-cell membranes via mRNA-LNP delivery would simultaneously block viral entry and re-educate adaptive immunity. The study compared three LNP mRNA vaccines that encode: (i) the small surface protein alone (S mRNA), (ii) the large + small proteins (LS mRNA, 1:1), and (iii) the full large + middle + small set (LMS mRNA, 1:1:1). In HBV-transgenic mice (high HBsAg load), after one priming dose, HBsAb appeared in 100% of high-dose LMS recipients, 80% of low-dose LMS, and 60%–80% of LS or S groups; by the third boost, LMS and LS drove HBsAb titers >4 × 10^4^ IU L^−1^, while S remained lower. In the pAAV-HBV1.2 model, the two 6–15 μg LMS shots cleared HBsAg, HBeAg, and HBV DNA within 5 weeks, generating earlier and higher HBsAb than S mRNA, which achieved similar Ag loss only by week 5 and with slower seroconversion. In the rAAV8-HBV1.3 model, all three vaccines lowered HBsAg and HBV DNA, but Ab hierarchy was LMS > LS ≫ S; LMS and LS also suppressed HBeAg in some mice, whereas S chiefly reduced Ag without robust HBsAb induction—implying a T cell-dominant effect. Thus, LMS delivered the most balanced humoral-and-cellular immunity and the fastest virological control, while LS matched its Ab potency with a simpler two-mRNA formulation. S mRNA alone excels at early HBsAg suppression—likely via stronger T cell priming—but fails to drive high-titer Abs. Nevertheless, several translational gaps remain in this study. Intra-hepatic cccDNA was not quantified, leaving true reservoir clearance unproven. Efficacy varied: HBeAg loss was incomplete, and transgenic models with high immune tolerance showed only moderate reductions in HBsAg. All data were obtained from small, single-species cohorts, with follow-up limited to 32 weeks. The use of three separate guanosine monophosphate (GMP)-grade mRNAs and proprietary LNPs raises concerns about scalability and cost. Furthermore, more extensive studies on toxicology, biodistribution, and durability in humanized liver or primate models are needed before clinical translation can proceed.^94^

In another study, the approach focused on activating cytotoxic CD8^+^ T cell responses, which are essential for the elimination of HBV-infected hepatocytes, though mRNA-LNP vaccines that progressively intensify class I Ag presentation.^95^ The baseline construct encoded the native surface protein (HBsAg mRNA-LNP). A second design inserted an N-terminal MHC I signal peptide (SP-HBsAg) to route nascent Ag into the endoplasmic reticulum/cross-presentation pathway, while a third variant added a C-terminal MITD to prolong peptide residency on H-2Kb/H-2Db molecules (SP-HBsAg-MITD). Alum-adjuvanted ENGERIX-B (recombinant HBsAg) served as the licensed comparator.^95^ In naive BALB/c and C57BL/6 mice, two doses of native HBsAg mRNA-LNP elicited geometric-mean anti-HBs titers of ≈1.8 × 10ˆ5 IU mL^−1^ and a balanced IgG2c/IgG1 profile, already surpassing ENGERIX-B by an order of magnitude. Adding the signal peptide preserved Ab magnitude but doubled the frequency of multifunctional (IFN-γ^+^/TNF-α^+^) CD8^+^ cells, whereas inclusion of MITD accelerated seroconversion (protective Ab after a single prime) and tripled the number of granzyme-B-positive CTLs.^95^ In an acute HBV-challenge model, SP-HBsAg-MITD conferred sterile protection in all animals, SP-HBsAg reduced viraemia to below assay limits in >80%, and native HBsAg protected 70%–80%; the protein vaccine allowed frequent breakthrough infection. Therapeutic efficacy was tested in rAAV8-HBV1.3 carriers. Three weekly injections drove serum HBsAg down by ≈ 2.4 log_10_ with the native construct, by 3.0 log_10_ with SP-HBsAg, and to undetectable levels in six of eight mice given SP-HBsAg-MITD; corresponding anti-HBs seroconversion rates were 50%, 63%, and 75%, respectively.^95^ The MITD-engineered vaccine also produced the deepest reduction in serum HBV DNA (≈3.1 log_10_) and the highest splenic pool of HBsAg-specific IFN-γ–secreting CD8^+^ T cells. By contrast, ENGERIX-B neither lowered antigenemia nor generated detectable T cell responses. Across regimens, body-weight curves, liver enzymes, and cytokine panels revealed no overt reactogenicity. Thus, stepwise augmentation of trafficking motifs validates the design logic: SP improves cross-presentation and Th1 bias, and MITD further amplifies cytotoxic T cell fitness and virological control. Despite promising results, key translational gaps remain. The AAV model lacks substantial authentic cccDNA, so true reservoir clearance was not evaluated. The durability of response beyond 10 weeks and potential viral rebound after therapy cessation were not assessed. Since all data come from mouse models, questions remain about dose scaling and innate cytokine-related safety in larger species. Additionally, the use of three distinct GMP-grade RNAs combined with proprietary SM-102 LNPs adds manufacturing complexity. Future studies should combine MITD-optimized mRNA with NAs and evaluate cccDNA endpoints in humanized liver or primate models to support clinical translation.^95^

In addition, preclinical trials highlight the use of mRNA to improve siRNA vaccines against HBV.^96^ There was an engineered LNP containing a pair of chemically modified siRNAs that together cover ≈98.6% of HBV genotypes and, in a second formulation, co-loaded the same tailored LNP (tLNP) with mouse IL-2 mRNA (tLNP/siHBV-IL-2) to couple RNA-interference-mediated Ag knock-down with cytokine-driven immune rescue.^96^ Five weekly intravenous doses of tLNP/siHBV in rAAV-HBV1.3 carrier mice suppressed serum HBsAg and HBV DNA by up to 3 log_10_ relative to PBS with no overt toxicity.^96^ When IL-2 mRNA was included, 4/8 mice (50%) maintained HBsAg <100 IU mL^−1^, 5/8 (62.5%) retained >2 log_10_ HBV-DNA reduction, and 2/8 (25%) seroconverted to anti-HBs; the composite particle showed ∼97% encapsulation efficiency, augmented HBV-specific CD4^+^/CD8^+^ T cell infiltration and effector activation, yet left intra-hepatic cccDNA unchanged.^96^ By pairing pan-genotypic siRNA with an immunostimulatory mRNA in a single SM102-based LNP, the work elegantly addresses viral escape and immune exhaustion, achieving one of the deepest (≥3-log) Ag-and-DNA knockdowns reported for an siRNA-only HBV therapy. Nonetheless, efficacy was heterogeneous, reservoir (cccDNA) clearance was not achieved, and the rAAV-HBV1.3 model lacks HBV integration, limiting translational certainty. Pharmacokinetic mismatch (week-long siRNA activity vs. ∼24 h IL-2 expression) and relatively high RNA doses further underscore the need for dose optimization, stability engineering, and validation in humanized-liver or primate systems before clinical advance.^96^ Also, Huang et al. developed a novel HBV S region-targeted GalNAc-conjugated siRNA (KC13-M2G2).^97^ *In vivo*, the GalNAc-conjugated siRNA KC13-M2G2 achieved very high antiviral efficacy in mouse models of chronic HBV (rAAV-HBV-B and -C mice); HBsAg and HBV DNA loss occurred in 100% and 90% of animals, respectively, with HBsAb seroconversion in 80% and 30%; in rAAV-HBV-D mice, 40%–60% of animals in the 0.3–3 mg/kg groups developed HBsAb seroconversion, and in a stringent rAAV-HBV-D model with high baseline HBsAg (∼20,000 IU/mL), 60% of mice maintained sustained loss of HBsAg and HBV DNA with parallel HBsAb seroconversion up to day 189. In HBV-transgenic mice, 3 weekly doses of 3 mg/kg induced HBsAg loss in ∼83%–100% (5–6/6) of animals between days 14–42, while a single 3 mg/kg dose produced HBsAg loss at nadir in 50% (6/12) of mice. By contrast, in Sprague-Dawley rats and cynomolgus monkeys, KC13-M2G2 has been evaluated only for safety—showing acceptable tolerability up to 200 mg/kg, with mainly mild, non-adverse hepatic vacuolation and a defined no-observed-adverse-effect level—but without any reported antiviral efficacy readouts.

In sum, HBV-targeted LNP mRNA platforms demonstrated that judicious Ag selection, intracellular-routing motifs, or combination with siRNA can (i) reactivate exhausted anti-viral T and B cells, (ii) drive large log-scale reductions in serum HBsAg and HBV DNA, and in the best case (full-length HBsAg mRNA) (iii) achieve a measurable cut in intra-hepatic cccDNA. Collectively, they demonstrate that mRNA and also siRNA technology enable the generation of both multi- and single-Ag constructs optimized for immune cross-presentation without exogenous adjuvants and may serve as the basis for both preventive vaccination and treatment of chronic HBV.

## Clinical trials of mRNA-based HBV vaccine

**Figure 5:**
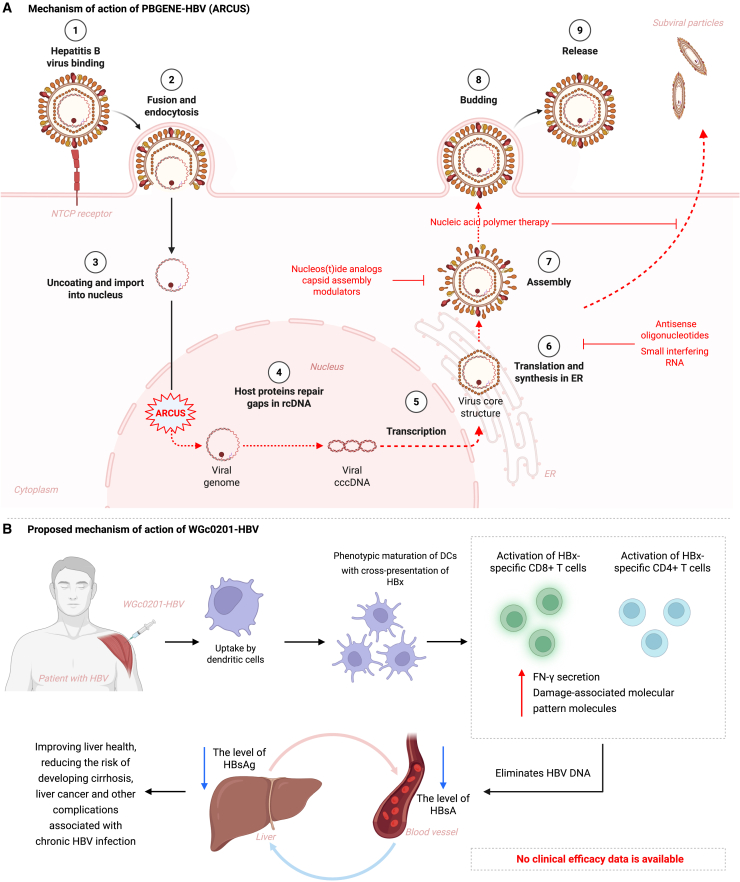
Mechanistic actions of two first-in-human mRNA therapeutics for chronic hepatitis B (A) PBGENE-HBV (ARCUS gene-editing mRNA). LNP-delivered mRNA is translated in hepatocytes into an ARCUS nuclease that enters the nucleus and cleaves both cccDNA and integrated HBV sequences, thereby eliminating the viral transcriptional template; ancillary red arrows indicate where existing NAs, capsid inhibitors, antisense/siRNA, or nucleotide-polymer agents act in the replication cycle. (B) WGc0201-HBV (HBx-encoding immunotherapy). After intramuscular injection, the HBx mRNA-LNP is taken up by dendritic cells, driving cross-presentation of HBx peptides, maturation of antigen-presenting cells, and activation of HBx-specific CD8^+^ and CD4^+^ T cells; the resulting IFN-γ-rich response lowers circulating and hepatic HBsAg, improves liver health, and reduces long-term risks of cirrhosis and HCC.

Several mRNA-encoded hepatitis B immunotherapies have progressed to first-in-human evaluation (Table 1). A prominent front-runner is PBGENE-HBV, which uses an LNP-delivered mRNA to express an ARCUS gene-editing nuclease that is tailor-engineered to excise cccDNA and inactivate integrated HBV DNA inside hepatocytes—a highly specific, potentially curative strategy aimed at achieving a functional cure (Figure 5A). The program received FDA Fast-Track designation in April 2025. Interim results from cohort 1 of the global Phase 1 ELIMINATE-B study (NCT06680232) were reported at ASGCT 2025. The lowest dose cohort, cohort 1 (0.2 mg/kg), involved only 3 patients, each receiving three administrations approximately 8 weeks apart. The patients enrolled exhibited varying baseline HBsAg levels, spanning a range from 562 to 11,813 IU/mL. Following treatment, the therapy demonstrated proof-of-activity by achieving substantial antiviral effects across all three participants. The best observed HBsAg reductions compared to baseline levels for 3 patients, respectively, were 56%, 69%, and 47%. Crucially, one of the 3 patients sustained an HBsAg reduction of approximately 50% (0.3 log) that was maintained 7 months after the initial dose administration, as of the data cutoff date of July 28, 2025. Safety data indicated that PBGENE-HBV was generally well-tolerated in cohort 1. No patient experienced a treatment-related adverse event above grade 2, nor were any serious adverse events or dose-limiting toxicities reported. Furthermore, no clinically significant abnormalities were detected in laboratory markers, including liver enzymes and platelets.^98^

**Table 1:** Current clinical trials of mRNA vaccine for HBV therapy in 2025

Name	PBGENE-HBV	WGc0201-HBV
Vaccine type	therapeutic	therapeutic
Encoding	LNP-mRNA encoding ARCUS meganuclease	LNP-mRNA encoding HBx antigen
Company or sponsor	Precision BioSciences, US	WestGene Biopharma, China
NCT/Phase	NCT06680232 (Phase 1, recruiting)	NCT07051187 (phase 1, not yet recruiting)
Status	cohort 1 complete; Cohort 2 dosing in progress; DMC endorsed enrolling cohort 3. Currently enrolling globally	not yet enrolling
Study period	2021–2026	2025–2027
Desing	part 1 is to identify a safe and well-tolerated dose regimen of PBGENE-HBV. Part 2 is an expansion cohort to aid in selecting a dosing regimen; experimental: participants in both parts 1 and 2 will receive a finite course of PBGENE-HBV; All participants will receive a finite course of multiple IV dose administrations of PBGENE-HBV. In Part 1, this will be done in a dose escalation manner, which may be evaluated further in a Part 2 expansion cohort	Wgc0201 will be administered by the intramuscular route, with a total of 9 doses
Dosing/Schedule	3-dose course. Starting from 0.2 mg/kg, intravenous. Three planned administrations, dosed approximately 8 weeks apart	9-dose course. A total of 9 doses planned. An estimated 9 patients, divided into 3 dose groups (3 patients per group), intramuscular
Population characteristics and eligibility	HBeAg-negative chronic HBV patients. Must be treated daily with nucleos(t)ide analog therapies; baseline HBsAg ≥200 IU/mL (no upper limit).	adult (18–65 years); chronic HBV (HBsAg positive >6 months). Received only NAs in the past 12 months and still taking them; HBV-DNA viral load below 100 IU/mL; HBsAg < 1500 IU/mL
Exclusion criteria	no history of cirrhosis of the liver; No current infections of hepatitis A, D, and E, human immunodeficiency virus (types 1 and 2), and no history of or current hepatitis C; No other active infections were deemed clinically relevant; No signs of hepatocellular carcinoma; Not received an organ transplant; No malignancy within 5 years of screening, except for specific cancers that are cured by surgical resection (e.g., basal cell skin cancer); No investigational agent received within 6 months of screening	HIV infection or HDV co-infection; Liver biopsy within 6 months showing cirrhosis or advanced fibrosis (Metavir A3/F3–F4; Ishak 4–6). If no biopsy: FibroScan >9 kPa or FibroTest >0.48 and APRI >1 (within 6 months); ALT >3× ULN; INR >1.5; Albumin <3.5 g/dL; Direct bilirubin >1.5× ULN; Platelet count <100,000/μL; History of hepatic decompensation or prior HCC; significant cardiovascular, respiratory, endocrine/metabolic, autoimmune, infectious, malignant, neurological, or psychiatric disease judged unsuitable by the investigator; Participation in another drug/device clinical trial within the past 3 months; History of organ transplantation (except corneal or hair transplantation); alcohol abuse (≥40 g/day for men or ≥20 g/day for women for >5 years) or known drug dependence; planned pregnancy, sperm/egg donation, or unwillingness to use effective contraception during the trial and for 6 months afterward
Primary endpoint	characterize the safety of PBGENE-HBV—the frequency of adverse events after 4 weeks of administration	observe and evaluate the safety of the vaccine, specifically the incidence and severity of adverse events at 12 weeks after the administration
Secondary endpoints	at 4 weeks: Pharmacokinetics; AUC: total PBGENE-HBV exposure over time; Cmax: time of maximum concentration; Cmin: minimum (trough) concentration; t½: terminal half-life; At 48 weeks: Frequency and severity of adverse events; physical exams, vital signs, and safety labs; antiviral activity; changes HBsAg and anti-HBs from baseline; Changes HBV DNA from baseline	at 12 weeks: Immunogenicity of the vaccine (HBV-specific T cell response), changes in virological indicators (HBV DNA/RNA), and changes in virological indicators (HBsAg), Changes in virological indicators (ALT normalization rate)
Expected completion	data update expected later in 2025. Phase 2 study expected to commence by H2 2027	not specified in sources
Results	proof of activity established. Substantial HBsAg reduction was observed in all 3 patients. Well-tolerated safety profile established	no efficacy data is available

Another potential mRNA-based vaccine against HBV is WGc0201-HBV—the HBx-encoding mRNA therapeutic vaccine. The LNP formulation delivers nucleoside-modified RNA that translates the hepatitis B oncoprotein HBx together with an “immuno-enhancer” sequence, driving potent Ag presentation and CD8^+^ T cell priming against HBV-infected or HBx-positive tumor cells (Figure 5B). No efficacy data is yet available for WGc0201-HBV. The trial (NCT07051187) is structured as an open-label, single-arm, dose-escalation phase I clinical trial. The total estimated enrollment is 9 patients, assigned to a single group where WGc0201 will be administered by the intramuscular route for a total of 9 doses. The phase I protocol utilizes 3 dose groups, each containing 3 patients, with the primary objective focused on observing and evaluating the safety of the vaccine via the incidence and severity of adverse events. Also, there is the clinical trial of WGc0201-HBV in combination with Tislelizumab for therapy HCC with high risk of recurrence and metastasis after radical therapy (NCT07077369). These clinical trials of WGc0201-HBV will clarify whether HBx-targeted mRNA immunotherapy can (i) reduce viral Ag load in chronic carriers and (ii) eradicate residual HBV-driven tumor cells when paired with checkpoint blockade. Early clinical read-outs are expected in 2026, positioning WGc0201-HBV as a complementary approach to gene-editing candidates such as PBGENE-HBV in the multi-modal quest for a functional cure.

Despite the potential of mRNA vaccines for therapy of HBV, therapeutic strategies are designed for achieving a functional cure, which is rigorously defined as HBsAg serological clearance (or levels below 0.05 IU/mL) coupled with sustained suppression of HBV DNA. The ultimate goal, however, remains a complete cure, necessitating the total eradication of all persistent viral DNA, specifically cccDNA and integrated HBV DNA. Trial design must account for the required study duration for durable off-treatment response; for combination therapies, efficacy endpoints are often measured 24–72 weeks after drug cessation to confirm sustained viral control.^99^ Since monotherapy is typically insufficient, trials rely on combination strategies, requiring careful comparator arm selection that frequently uses a backbone of NA therapy. Crucial patient stratification strategies prioritize enrolling patients with favorable prognostic markers, leveraging the fact that lower baseline serum HBsAg levels significantly predict better outcomes. For instance, the phase I WGc0201 trial specifically enrolls patients already stabilized on NAs with HBV-DNA below 100 IU/mL and a relatively low Ag burden of HBsAg less than 1500 IU/mL. This approach supports a proposed stepwise treatment scheme where sustained Ag downregulation precedes the boosting of host antiviral immune responses.^99^

In summary, therapeutic HBV mRNA vaccines have produced striking proof-of-concept results in animal models, with sustained reductions in HBsAg and restoration of HBV-specific CD4^+^ and CD8^+^ T cell responses that clearly surpass those achieved with conventional protein vaccines. In contrast, established prophylactic subunit vaccines, based on yeast-derived HBsAg adsorbed to alum, excel at inducing high-titer neutralizing anti-HBs antibodies and long-lived humoral memory in naive individuals, but they generate relatively weak cytotoxic T cell responses and have shown little ability to reset the profoundly tolerant T cell compartment of chronic HBV infection. By design, the newer therapeutic platforms—DNA vaccines, viral vectors, and mRNA-LNP constructs—deliver Ags endogenously within host cells so that they are processed through MHC class I and II pathways, mimicking acute infection and driving strong CTL and polyfunctional T cell responses. However, clinical experience to date illustrates the difficulty of this task: plasmid DNA vaccines and adenoviral or modified vaccinia ankara (MVA) vectors can boost HBV-specific T cells in virally suppressed patients, yet have so far produced only modest and infrequent declines in virological markers and have not consistently prevented post-treatment rebound, underscoring the challenge of overcoming entrenched intrahepatic tolerance and clearing cells that harbor cccDNA. mRNA vaccines occupy an intermediate position in this landscape (Table 2). Like DNA and viral vectors, they function as sophisticated intracellular Ag-delivery systems intended to make the immune system perceive chronic HBV as a *de novo* acute infection, but they do so without nuclear entry or vector persistence, and with a built-in innate adjuvant effect from the RNA and LNP that can be tuned by sequence and formulation engineering (Table 2). Preclinical data suggest that this combination of intense but transient Ag expression and strong innate stimulation can break functional tolerance and, in some models, eliminate detectable antigenemia. Whether these advantages will translate into clinically meaningful rates of sustained HBsAg loss or functional cure in chronically infected patients remains unknown. It is likely that therapeutic mRNA vaccines will ultimately be deployed in rational combinations—with potent viral-load–lowering agents such as NAs or siRNA, and possibly with immune checkpoint inhibitors or other immunomodulators—to maximize the probability of durable immune control and minimize the risk of hepatic flares. Comparative assessment of subunit, DNA, mRNA, and viral-vector platforms (Table 2) indicates that mRNA-based immunotherapies currently represent the most flexible and immunogenically powerful, yet clinically unproven, approach in the quest for a functional cure for chronic HBV.^99^

**Table 2:** Comparison of HBV vaccine platforms with prophylactic and therapeutic focus

Platform type	Subunit/Adjuvant (established recombinant prophylaxis, e.g., yeast-derived HBsAg)	DNA vaccines	mRNA vaccines	Viral vectors (adenovirus, MVA, etc.)
Mechanism of action	uses recombinant HBsAg (often from *S. cerevisiae*) that self-assembles into non-infectious virus-like particles.^100^ Antigen is adsorbed onto aluminum salts (alum), which stabilizes the protein and acts as a Th2-biased adjuvant.^101^ The alum-HBsAg complex is taken up by antigen-presenting cells for processing	plasmid DNA encoding HBV antigens (e.g., HBsAg, HBcAg) are delivered into host cells, where they are transported to the nucleus and transcribed into mRNA. The mRNA is then translated in the cytoplasm to produce viral proteins that are presented on MHC I and II, mimicking natural infection.^102^ This endogenous antigen expression drives both antibody and T cell responses	LNP-encapsulated mRNA encoding HBV antigens (e.g., HBsAg subunits) is delivered into host cells, where it is translated directly in the cytoplasm into viral proteins.^95^ The mRNA itself (especially if nucleoside-modified) also acts as a strong innate stimulus via RNA-sensing pathways, enhancing immune activation. There is no risk of genomic integration with mRNA	replication-deficient viral vectors (e.g., AdV5, MVA) are engineered to carry HBV genes (core, polymerase, and surface proteins). Upon vaccination, the vectors infect host cells and express these HBV antigens intracellularly. Heterologous prime-boost regimens (e.g., DNA prime/MVA boost or Adeno prime/MVA boost) are often used to maximize immunity
Immune response profile	elicits very strong anti-HBs antibody responses, which correlate with protection.^100^ Generates durable humoral immunity and immunological memory; vaccinated individuals remain protected via memory B and T cells that can rapidly recall an immune response. Alum skews the response toward Th2-type immunity, producing high antibody titers but limited cytotoxic (Th1/CTL) stimulation^101^	aims to induce both humoral (Abs) and strong cellular immunity. By driving intracellular antigen synthesis, DNA vaccines efficiently stimulate CD8^+^ cytotoxic T lymphocytes (via MHC-I) as well as CD4^+^ helper T cells.^102^ In preclinical models, DNA vaccines for HBV elicited robust CTL and antibody responses in mice and primates^102^	induces potent innate and adaptive immunity: LNPs provoke innate sensors, and the antigen expression generates high titers of anti-HBs antibodies and robust CD4^+^ and CD8^+^ T cell responses.^95^ In animal models, HBsAg mRNA-LNP vaccines produced stronger humoral and cellular responses than protein vaccines. In a chronic HBV mouse model, mRNA vaccination led to antigen clearance and strong T cell activation^95^	elicit strong innate and adaptive immunity: viral proteins stimulate dendritic cells and robust polyfunctional T cell responses. Vaccination induces HBV-specific CD4^+^ and CD8^+^ T-cells that secrete cytokines (IFN-γ, TNF-α, IL-2) upon antigen encounter.^103^ For example, an Ad5-based TG1050 vaccine in humans elicited IFN-γ-producing T cells against HBV core/polymerase/surface. In nonhuman primates, an Ad-HBV vector induced broad IFN-γ and IL-2 T cell responses to multiple HBV epitopes^103^
Manufacturing scalability	produced by recombinant DNA technology in yeast, allowing large-scale, high-yield antigen production.^100^ Established manufacturing pipelines exist for yeast-derived HBsAg	plasmids are produced in bacteria, allowing cost-effective and scalable fermentation. DNA vaccine products are highly stable: they do not require a stringent cold chain and can be lyophilized. (For example, studies note DNA vaccines do not require cold storage and are stable at room temperature.)	mRNA vaccines are synthesized by cell-free *in vitro* transcription, enabling very rapid and scalable production once the sequence is defined.^104^ Production does not require cell cultures or fermentation. This “plug-and-play” manufacturing allows quick redesign for different antigens	adenovirus and MVA production are well established (e.g., cell culture bioreactors), but scaling to global demand is complex. Both vector types require cell substrate production and purification, which can be scaled industrially, though with higher cost and complexity than subunit or nucleic vaccines
Cold chain requirements	requires standard refrigeration (2°C–8°C); freezing must be avoided to prevent antigen detachment from alum.^100^ HBV subunit vaccines have shown relative heat stability in WHO studies, enabling controlled-temperature-chain (CTC) use for limited excursions (e.g., up to ∼1 month at ≤37°C)^100^	generally stable at ambient temperature (no live organism); standard nucleic acid vaccines do not need deep freeze. Cold storage is not required as for live vaccines. Exact packaging/transport conditions depend on formulation^102^	typically requires frozen or ultra-cold storage to maintain mRNA integrity (as seen in approved COVID-19 mRNA vaccines). The RNA is inherently labile, so cold-chain logistics must be carefully managed.^104^ Specific data for HBV mRNA vaccines are not yet established, but similar cold-chain demands are expected	viral vector vaccines generally require refrigerated storage (≈2°C–8°C); exact requirements depend on formulation. (Most adenoviral/MVA vaccines are stable at 2°C–8°C for months.) Specific cold-chain data for experimental HBV vectors are not widely reported, but they likely follow standard vaccine storage guidelines
Prior clinical outcomes	highly effective prophylaxis: induces seroprotective anti-HBs in >95% of healthy vaccinees. Confers long-term protection (effective immunity lasting 20–30+ years) in immunocompetent individuals. New adjuvanted formulations (e.g., with toll-like receptor agonists) further improve responses in older or hypo-responder adults^100^	to date, therapeutic efficacy in humans has been limited. For example, a Phase I/II trial of a DNA vaccine added to NAs (after ∼3 years of suppression) showed no improvement in sustained viral control: HBV relapse occurred in ∼97% of patients regardless of vaccination, and no augmented antiviral immunity was seen.^105^ Some HBV-specific CD4^+^ T cell responses were induced, but these did not translate into clinical benefit^105^	preclinical results are promising. E.g., a 2025 study showed that HBsAg mRNA-LNPs induced “robust humoral and cellular responses, outperforming the protein vaccine” and cleared antigen in chronically infected mice.^95^ However, clinical data in humans are still early, and no therapeutic mRNA HBV vaccine has yet demonstrated clear efficacy in chronic HBV patients^100^	preclinical studies, e.g., TG1050 (Adeno-5) in HBV-carrier mice, reduced viral markers and induced HBV-specific T cells. Clinical trials: Phase 1b TG1050 was safe and induced HBV-specific T cell responses (measured by IFN-γ ELISPOT) in suppressed CHB patients.^103^ Some subjects showed modest declines in HBV RNA or HBcrAg. Overall, T cell immunity is boosted, but full virus clearance was not achieved

## Reasons for the failure of therapeutic vaccines

Therapeutic vaccines for chronic HBV have repeatedly underperformed because they are trying to reboot an immune system that is fundamentally “broken” by chronic infection. In long-standing HBV, high and continuous antigenemia (especially HBsAg) and intrahepatic expression of viral proteins drive profound CD4^+^/CD8^+^ T cell exhaustion, expansion of regulatory/suppressor cells, and up-regulation of inhibitory receptors, so that vaccine Ags are presented into a highly tolerant, immunosuppressive milieu. Reviews of protein, DNA, and viral-vector vaccines show that, although many candidates (e.g., yeast-derived HBsAg, DNA vaccines, and adenoviral TG1050) can boost HBV-specific T cells or antibodies, they usually produce only modest, transient declines in HBsAg or HBV DNA and almost no off-treatment functional cure, because they neither sufficiently reduce viral Ag load nor eliminate cccDNA/integrated DNA reservoirs that continuously reseed tolerance and viremia.^106^^,^^107^^,^^108^ mRNA platforms address several of these weaknesses: in contrast to DNA and many viral vectors, *in vitro*-transcribed mRNA functions entirely in the cytoplasm, avoiding nuclear entry and insertional risk; it is rapidly designed and manufactured; and, when formulated in lipid nanoparticles, it drives high-level, transient Ag expression with efficient MHC-I and MHC-II presentation, strong CD4^+^/CD8^+^ responses, and tunable innate adjuvanticity without exogenous adjuvants.^109^^,^^110^^,^^111^^,^^112^ In HBV models, mRNA–LNP vaccines encoding full-length HBsAg or multi-Ag constructs have achieved complete HBsAg clearance and multi-log HBV-DNA reductions in carrier mice, and the first human trials now combine mRNA-encoded nucleases or HBx vaccines with NAs to attack both the immune defect and the nuclear reservoir. However, several barriers remain common to all therapeutic platforms, including mRNA: deep intrahepatic immune tolerance that is not fully reversed by vaccination alone; persistence and epigenetic resilience of cccDNA and integrated HBV DNA; viral genotypic and epitope diversity; imperfect animal models that over-predict “cure”; and practical issues such as reactogenicity from innate RNA sensing, cold-chain constraints for LNP formulations, and the need for long, combination-therapy trials with off-treatment follow-up.^113^^,^^114^^,^^115^ Consequently, the field increasingly views mRNA not as a stand-alone solution but as a flexible backbone for combination regimens (with NAs, siRNA, antisense oligonucleotide (ASO), checkpoint blockade, or gene editing) designed to first debulk Ag and then use mRNA’s immunologic strengths to break tolerance and sustain a true functional cure.

## Challenges of mRNA-based hepatitis prevention and therapies

### Ag selection amid viral diversity

HBV presents 10 genotypes (A–J) that show distinct geographic and ethnic distributions, influencing disease progression, response to therapy, and vaccine efficacy.^8^^,^^26^^,^^44^ Also, there are >40 sub-genotypes and a growing catalog of immune-escape mutations in the S (“a” determinant), core, and X proteins.^40^^,^^41^ Choosing which open reading frames to encode is therefore non-trivial: monovalent HBsAg mRNA may be thwarted by vaccine-escape variants, whereas adding core, polymerase, or HBx sequences broadens T cell coverage but enlarges the transcript and can dilute expression. Head-to-head mouse work confirms that multi-Ag constructs (core + pol + preS2-S) elicit deeper HBsAg declines than HBsAg alone but also trigger stronger innate sensing that must be tamed by nucleoside modifications and sequence engineering.^28^^,^^93^

### Manufacturing and cost

The production of mRNA vaccines for HBV faces significant cost and complexity challenges. Manufacturing must comply with strict GMP standards, involving expensive enzymes, nucleotides, and purification processes. For example, conventional *in vitro* transcription methods required a separate enzymatic capping step that was inefficient and relied on costly enzymes.^116^ Newer techniques (such as co-transcriptional capping with cap analogues) are helping to reduce these expenses, but the overall cost of goods remains high for mRNA-based products. Formulating the mRNA into LNPs adds another layer of complexity. LNP production demands sophisticated mixing equipment and tight process controls to ensure particle uniformity and potency. Each batch must undergo rigorous quality testing, contributing to higher production costs than traditional protein vaccines.

In addition, mRNA-LNP vaccines currently require ultra-cold storage to maintain stability. It is needed because mRNA is produced in a cell-free, enzymatic *in vitro* transcription workflow, yet today’s LNP formulations still depend on −20°C to −70°C distribution.^117^ Cold-chain fragility is a major impediment to global HBV immunization campaigns. For example, lyophilization and revised lipid chemistries can stabilize LNPs for months at 4°C but require new fill-finish infrastructure and regulatory bridging.^118^^,^^119^ It was shown *in vivo* that <2% of internalized LNP-mRNA escapes the endosome^118^^,^^119^^,^^120^^,^^121^; chemical fusogens and ionizable lipids are being iterated to raise that figure, but every tweak demands renewed toxicology, delaying scale-up. To solve the limitation, there is a protocol for the production of mRNA in a cell-free, enzymatic *in vitro* transcription workflow. This was exemplified during the coronavirus disease 2019 (COVID-19) vaccine rollout, where many low- and middle-income countries struggled to deploy mRNA vaccines due to cold chain limitations.^122^ These hurdles raise concerns about the feasibility of delivering an HBV therapeutic vaccine to regions where infrastructure is limited. Efforts are underway to mitigate these issues—for instance, through lyophilization techniques that render mRNA vaccines stable at standard refrigerator temperatures^122^—but until such innovations mature and become widely available, the high manufacturing costs and complex distribution requirements of mRNA vaccines remain a barrier to global access in chronic HBV therapy.

Thus, despite these manufacturing and stability constraints, the underlying production pathway of mRNA vaccines remains conceptually straightforward at the research and pre-clinical scale. A standardized end-to-end workflow is typically followed: (i) rational design of the Ag and untranslated regions; (ii) generation and linearization of a DNA template; (iii) high-yield *in vitro* transcription with co-transcriptional capping and nucleotide modification—steps that become increasingly expensive and tightly regulated when scaled under GMP; (iv) purification of the RNA and encapsulation into LNPs, a stage that mirrors the technical bottlenecks seen in commercial manufacturing due to stringent quality control and specialized equipment requirements; and (v) functional validation through transfection of cultured cells to confirm expression and potency.^123^ This streamlined laboratory workflow underscores the contrast between the relative simplicity of small-scale mRNA vaccine production and the substantial cost, process-control burden, and logistical complexities that emerge when these same steps are expanded to clinical-grade volumes for global HBV vaccine deployment.

### Global health considerations

The promise of an mRNA-based cure for HBV will mean little if it cannot reach the populations that need it most. HBV is a global disease with a disproportionate burden in resource-limited regions: of the ∼296 million people living with chronic hepatitis B, the majority reside in sub-Saharan Africa and East Asia.^124^ These regions often face fragile healthcare infrastructure, making the deployment of a complex vaccine therapy particularly challenging. For instance, many endemic countries still struggle with basic HBV control measures—birth-dose vaccinations, routine immunization, and antiviral therapy coverage—due to limited funds and healthcare access. Chronic HBV is severely underdiagnosed and undertreated worldwide, and this gap is even more pronounced in low-income countries.^124^ In such settings, identifying patients who would benefit from a therapeutic vaccine and delivering a multi-dose regimen safely will require significant investments in healthcare delivery systems. Without improvements like thermostable vaccine formulations or novel distribution models, there is a risk that an HBV mRNA vaccine would be largely confined to wealthy or urban centers, widening global disparities in HBV outcomes. Equity in access is a central concern—the experience during the COVID-19 pandemic, where low-income regions had delayed and limited access to mRNA vaccines, serves as a cautionary tale.^122^ To avoid a similar “vaccine apartheid” with HBV, global health strategies must proactively address affordability and infrastructure. This could involve tiered pricing, technology transfer (to enable regional vaccine production hubs), and international support for strengthening cold-chain and delivery networks. Initiatives like the WHO’s HBV elimination program and new public–private partnerships will be critical in ensuring that a cure, once available, does not remain the privilege of a few. Thus, the feasibility of an mRNA HBV vaccine in high-burden countries will depend on coupling the scientific advance with substantial improvements in healthcare infrastructure, financing, and political commitment to achieve equitable implementation.

### Delivery efficiency

Efficient delivery of mRNA to the appropriate cells is critical for a therapeutic HBV vaccine, yet current delivery systems have suboptimal intracellular release. After intramuscular or intradermal injection, mRNA-LNPs are taken up by cells (particularly Ag-presenting cells), but only a small fraction of the internalized mRNA escapes the endosomal compartment into the cytosol. Indeed, limited endosomal escape is widely regarded as the rate-limiting step for RNA therapeutics.^125^ Most of the mRNA remains sequestered or gets degraded in endolysosomal vesicles, which in turn restricts Ag expression and blunts the immune response. This inefficiency means that higher doses or repeated injections might be needed to achieve a therapeutic effect, potentially increasing the risk of reactogenicity. Emerging strategies are focusing on improving endosomal escape and overall delivery efficiency. One approach is the design of novel ionizable lipids and LNP formulations that become more disruptive in the acidic environment of endosomes, thereby facilitating cytosolic release of mRNA. For example, a recent study engineered “endosomolytic” LNPs by incorporating a chloroquine-mimicking moiety into the lipid structure, which significantly enhanced endosomal release.^126^ The resulting nanoparticles achieved up to a ∼19-fold higher *in vitro* transfection efficiency compared to conventional LNPs,^126^ and demonstrated improved mRNA delivery *in vivo*. Other approaches include incorporating pH-sensitive or fusogenic peptides and biodegradable polymers and employing high-throughput combinatorial screening to discover formulations with superior cellular uptake and release profiles.^125^ While these innovations are still largely in the experimental stage, they highlight potential solutions to overcome the endosomal bottleneck. Improving the efficiency of mRNA delivery will be pivotal for an HBV vaccine’s success, since even incremental gains in cytosolic delivery could translate to substantially stronger Ag expression and immune priming at a given dose.

### Safety and immune-response balance

mRNA strands and their carriers are intrinsically immunostimulatory—a double-edged sword. Moderate activation of RIG-I, TLR7/8, and STING boosts adjuvanticity, yet excessive type-I-IFN signaling can shut down translation and provoke flares in HBV-infected livers already primed for inflammation. Rare myocarditis and anaphylaxis signals seen with COVID-19 mRNA vaccines highlight the need for genotype-agnostic safety monitoring when HBV trials scale to thousands.^127^^,^^128^ Moreover, therapeutic vaccination may precipitate hepatic flares or HBV reactivation if given with checkpoint inhibitors or other immunosuppressants, demanding careful antiviral prophylaxis and ALT surveillance during combination regimens.^129^

### Durability off-treatment response

A therapeutic HBV vaccine must not only trigger an initial immune response but also sustain that response long enough to control or clear the infection. The durability of vaccine-induced immunity is therefore also one of the major concerns. Chronic HBV infection is characterized by high levels of viral Ags (such as HBsAg) that drive T cell exhaustion and dysfunction.^97^ Even if an mRNA vaccine can transiently invigorate HBV-specific T cells or induce neutralizing antibodies, the risk is that these responses could wane once the initial vaccine stimulus subsides, especially if high Ag loads persist. For example, in the context of therapeutic vaccination, a functional cure is usually defined by a durable off-treatment response—sustained loss of HBsAg and undetectable HBV DNA for 6–12 months after therapy. If the immunity provoked by the vaccine is not long-lasting, there is a possibility of viral rebound after the course of vaccination is completed. Lessons from other therapeutic approaches underscore this risk: patients on novel HBV drugs often experience a return of viral replication markers when treatment is stopped.^130^ In one example, discontinuation of experimental agents like capsid assembly modulators led to HBV RNA and Ag re-emergence, highlighting the virus’s ability to recrudesce if immune pressure is not maintained.^130^ Thus, a key challenge for an mRNA HBV vaccine is ensuring that it elicits robust memory T cell and B cell responses that can persist long-term. This might require multiple booster injections or combination with antivirals to keep the virus suppressed until vaccine-induced immunity fully matures. It will be crucial to monitor patients for several years in clinical trials to confirm that responses are durable and that HBsAg seroconversion (or other markers of immune control) remains stable. Ultimately, the goal is to induce immunity that does not just temporarily reduce viral load but permanently recalibrates the host-virus equilibrium in favor of the host. Achieving such lasting immune control—essentially retraining the immune system to keep HBV in check—remains one of the most formidable challenges for any curative HBV strategy.

### Regulatory pathway and market access

The regulatory and market landscape for an HBV therapeutic vaccine is uncharted territory. No precedent exists of an mRNA vaccine being approved to treat a chronic infection, which means regulatory agencies will have to define new benchmarks for efficacy and safety. Key efficacy endpoints are likely to include functional cure rates, such as sustained HBsAg loss and HBV DNA suppression after a period off therapy, as these correlate with improved clinical outcomes. Regulators will expect robust evidence that the vaccine can achieve a meaningful cure rate above and beyond what is possible with existing treatments. This is a high bar, given that even the most advanced new HBV therapeutics in trials (for example, antisense oligonucleotides and siRNA agents) have so far attained functional cure in only ∼10%–20% of patients.^97^ Demonstrating substantially higher cure rates, or the ability to synergize with oral antivirals to reach those cure rates, will likely be necessary for approval. Safety will also be scrutinized: a therapeutic vaccine must show that it can stimulate the immune system without causing severe flares of hepatitis or autoimmune side effects. Regulatory guidance for therapeutic vaccines is still evolving, but it may draw on elements from both vaccine and antiviral drug pathways, requiring large controlled trials with hard clinical endpoints (e.g., prevention of cirrhosis or HCC in addition to virological endpoints) and long follow-up. Market access considerations are equally critical. The cost and pricing of an mRNA HBV vaccine will determine how widely it is adopted in real-world healthcare systems. Currently, the standard of care for chronic HBV—NAs antivirals like tenofovir or entecavir—is available as low-cost generics in many countries, often costing only a few hundred dollars per patient-year. These drugs effectively suppress the virus, although they seldom cure it, and they must be taken indefinitely. In contrast, a short-course curative therapy, if successful, could obviate the need for lifetime treatment and long-term monitoring of viremia. Health economists will weigh the upfront price of the vaccine against the cumulative cost of decades of antiviral therapy and the potential costs of managing liver cirrhosis or cancer that a cure might avert. Models suggest that broad HBV treatment strategies can be highly cost-effective, even cost-saving, if drug prices are kept low.^131^ This implies that if a new vaccine is priced too high, its cost-effectiveness advantage over generic antivirals diminishes. Payers and national health systems may be reluctant to cover an expensive HBV vaccine unless the clinical benefit—in terms of cured patients and prevented complications—clearly justifies the expenditure. Therefore, manufacturers will likely face pressure to set pricing in line with value-based considerations, possibly adjusting for different markets. Another aspect of market access is reimbursement and health technology assessment: agencies will examine the quality-adjusted life years (QALYs) gained by curing HBV versus managing it chronically. If the vaccine demonstrates a chance at a true cure, it could be deemed cost-effective even at a relatively high price point, as was the case for curative hepatitis C antivirals, but this will depend on the cure fraction achieved and the price per course. Finally, to maximize public health impact, strategies such as advance market commitments, inclusion in global procurement schemes (*e.g*., the Vaccine Alliance Gavi for eligible countries), and academic–industry partnerships for technology transfer might be needed.^124^

## Conclusion and future perspectives

Chronic HBV remains notoriously difficult to cure with existing drugs. Amid this challenge, recent progress in mRNA-based therapies offers new hope. Because the transcript functions in the cytoplasm, any encoded protein or peptide can be generated efficiently by the cell’s own translation machinery in sustained protein output.^132^ Moreover, this cytoplasmic activity also gives mRNA higher transfection efficiency and lower toxicity than DNA, which must cross the nuclear envelope.^133^ Lacking genomic integration capacity, mRNA avoids the dangers of infection or insertional mutagenesis.^30^ Collectively, these advantages have accelerated the spread of mRNA platforms throughout biomedicine and opened new therapeutic avenues for disorders once deemed untreatable. Also, there are novel vaccine manufacturing technologies such as an on-chip microfluidic system that can synthesize and encapsulate mRNA–LNP vaccines *de novo* on demand.^134^ LNP formulations continue to evolve—new ionizable lipids and biodegradable or receptor-targeted LNPs are being designed to improve tissue targeting and minimize side effects. For example, researchers are adding targeting moieties (peptides, glycans) to LNP surfaces to direct mRNA vaccines to specific organs or cell types.^135^ Additionally*,* novel LNP engineering strategies have achieved organ-specific mRNA delivery by modulating lipid composition, enabling precise hepatic or pulmonary targeting.^136^ Such next-generation LNPs, combined with optimized mRNA chemistry (e.g., nucleoside modifications, sequence engineering), promise to maximize protein translation in hepatocytes while reducing off-target and innate immune activation, thereby boosting efficacy and safety of mRNA HBV therapies. For instance, an HBV mRNA vaccine optimized via an AI-guided codon optimization and pseudouridine substitution strategy showed greatly enhanced mRNA stability and protein expression in preclinical studies.^136^

In addition to this, there is a novel emerging approach—circular mRNA and self-amplifying mRNA vaccines. Circular mRNA is covalently closed RNA rings that resist exonuclease degradation and support extended protein expression, translating into more durable immunity.^137^ Meanwhile, self-amplifying mRNA vaccine platforms can drive prolonged Ag production and potent responses at much lower doses than conventional mRNAs.^138^ This dose-sparing effect is accompanied by intrinsic adjuvanticity from replicative RNA species, although careful design is required to manage reactogenicity. These advances could enable personalized, point-of-care production of mRNA vaccines, ensuring that combination immunotherapies can be rapidly customized to individual patients in the future.

Beyond mRNA and delivery optimizations, exploiting other biological platforms is an active area to improve HBV vaccine responses. In additional new strategies, microbial-based strategies are being explored to modulate immunity against HBV. For instance, there is growing evidence that the gut–liver axis can be leveraged—administering probiotic bacteria has been shown to enhance antiviral T cell activity.^139^ For example, a probiotic (*Bifidobacterium longum*, *Lactobacillus acidophilus*, and *Enterococcus faecalis*) with a metabolite, spermidine, significantly augmented HBV-specific -γ+CD4+ T cell immunity to inhibit HBV and accelerated the decline of serum HBsAg in treated mice and patients on antivirals.^139^ These findings suggest that engineered bacterial carriers or their metabolites could serve as adjuvants to overcome immune exhaustion in chronic HBV. Likewise, viral vectors are being harnessed as vaccine vehicles to break tolerance.^140^ A notable example is the TherVacB approach (German Center for Infection Research), which employs a heterologous prime–boost—a priming with HBV protein Ags followed by a Modified Vaccinia Ankara viral vector boost encoding HBV Ags to robustly activate T cells. This strategy aims to drive both potent CD8^+^ responses and neutralizing Abs against HBV. Similarly, other recombinant viral platforms (e.g., adenoviral vectors) encoding HBV Ags have demonstrated the ability to lower HBsAg in chronic carriers in early trials.^141^^,^^142^ These complementary approaches—from leveraging commensal microbes to viral vectors—underscore a broader paradigm of enlisting live biological systems to stimulate the host immune response against HBV. In the future such modalities might be combined with mRNA vaccines to synergistically improve therapeutic vaccine efficacy.

Presumably, future HBV therapies are converging on combination regimens that target the virus on multiple fronts. A functional cure (sustained off-treatment remission) will likely require both drastically reducing the viral burden and reawakening antiviral immunity.^143^ For example, an mRNA-based approach could be paired with RNA-silencing therapies (siRNAs or antisense oligonucleotides) to potently shut down HBV gene expression, while patients remain on NAs to keep viral DNA low.^143^ By sharply lowering circulating HBsAg and HBV DNA, these combinations can alleviate T cell exhaustion, creating a window in which immunotherapeutic vaccines may work more effectively. One notable example is Vir Biotechnology’s MARCH trial, which combines a subcutaneously delivered HBV-targeted siRNA elebsiran (VIR-2218) with a broadly neutralizing anti-HBsAg Ab tobevibart (VIR-3434)—with some study arms also including PEG-IFNα. This triple approach aims to simultaneously reduce viral Ags and invigorate immune responses. Interim Phase 2 results showed that a subset of patients (especially those starting with lower HBsAg levels) achieved sustained HBsAg loss off treatment. However, overall cure rates remained modest, reinforcing that siRNA or ASO monotherapy achieves functional cure in only a minority of patients (on the order of ∼10%–20%).^143^ Another strategy under evaluation is sequential therapy that “debulks” the virus first, then boosts immunity. For instance, one clinical trial evaluated therapeutic vaccine platforms together with immune checkpoint inhibitors (e.g., PD-1 blockade) or with cytokine therapy (e.g., PEG-IFN-α) to rejuvenate HBV-specific T cells. Early data indicate that such combinations can induce HBsAg loss in a subset of patients—especially those with lower baseline Ag loads or less exhausted immune profiles.^144^

Notably, the vaccine induced HBV-specific T cells and was associated with HBsAg declines in patients with baseline titers below ∼100 IU/mL, and the addition of checkpoint blockade further enhanced immune responses in some cases.^87^ These early data underscore that baseline Ag load and T cell exhaustion status are key determinants of response—patients with lower HBsAg and less exhausted T cells respond best to immune-based therapies. In line with this, GSK’s B-Clear trial of the antisense oligonucleotide bepirovirsen reported that ∼9%–10% of chronically infected patients achieved off-treatment HBsAg clearance, almost exclusively among those with low pre-treatment HBsAg (<3,000 IU/mL).^145^ A follow-up study adding PEG-IFNα after bepirovirsen (the B-Together trial) yielded a similar functional cure rate (∼15% in the best arm) but with fewer post-treatment relapses, suggesting that sequential Ag knockdown followed by immune stimulation can improve the durability of remission.^143^ These examples illustrate a broader trend: multi-modal combinations—antiviral knockdown agents plus therapeutic vaccination (and sometimes immunotherapy boosters like cytokines or checkpoint inhibitors)—are being rationally designed to overcome HBV persistence.

Looking ahead, rationally designed combination regimens of mRNA vaccines with other therapeutic methods hold great promise to finally achieve durable HBV cures.

Notwithstanding this optimism, significant translational and regulatory hurdles must be overcome to realize these advances in the clinic. mRNA vaccines and gene therapies for HBV will require rigorous validation of safety and long-term efficacy. For instance, genome-editing candidates like PBGENE-HBV raise important regulatory considerations around off-target cleavage and potential genomic integration, necessitating comprehensive preclinical safety profiling and sustained patient monitoring in trials. Likewise, immune-focused therapies must carefully balance efficacy with the risk of immunopathology; a powerful T cell response could trigger hepatic flares, so dosing and patient selection will be critical. Many patients with chronic HBV have profoundly exhausted or deleted HBV-specific T cells after years of infection, posing a challenge for immunotherapies—especially in those with high Ag load or older age, who tend not to respond as well.^144^ Overcoming this may require high-cost patient-tailored approaches or adjunctive therapies to bolster immune responsiveness. From a regulatory perspective, demonstrating a “functional cure” is complex since it requires showing that HBsAg remains undetectable well after stopping therapy. Trial designs thus need prolonged follow-up and clear endpoints acceptable to agencies. Moreover, as these are first-in-class modalities for HBV, global regulatory agencies are still developing guidelines for manufacturing quality, clinical trial oversight, and post-marketing surveillance. This ongoing framework development contributes to the likelihood of a decade-long or longer timeline from Phase I to potential approval.

Despite these challenges, the coming years will be crucial for translating these cutting-edge therapies from bench to bedside, ultimately improving outcomes for millions of patients living with chronic hepatitis B.

## Acknowledgments

Nadezhda Pechnikova would like to express her deepest gratitude to her family and her friends for their unwavering love, patience, and support throughout this work.

## Author contributions

N.A.P., M.Z.-C., M.P., C.P., and I.I.: writing (original draft); Y.V.O. and A.V.Y.: writing (review and editing). All authors approved the final version of the manuscript.

## Declaration of interests

The authors declare no competing interests.
